# Effect of Germination and Illumination on Melatonin and Its Metabolites, Phenolic Content, and Antioxidant Activity in Mung Bean Sprouts

**DOI:** 10.3390/plants11212990

**Published:** 2022-11-06

**Authors:** Pimolwan Siriparu, Panyada Panyatip, Thanawat Pota, Juthamat Ratha, Chawalit Yongram, Tarapong Srisongkram, Bunleu Sungthong, Ploenthip Puthongking

**Affiliations:** 1 Master of Sciences Program in Pharmaceutical Chemistry and Natural Products, Faculty of Pharmaceutical Sciences, Khon Kaen University, Khon Kaen 40002, Thailand; 2 Department of Pharmacognosy, Faculty of Pharmacy, Srinakharinwirot University, Nakhon Nayok 26120, Thailand; 3 Melatonin Research Group, Khon Kaen University, Khon Kaen 40002, Thailand; 4 Division of Cannabis Health Science, College of Allied Health Sciences, Suansunandha Rajabhat University, Samut Songkhram 75000, Thailand; 5 Division of Pharmaceutical Chemistry, Faculty of Pharmaceutical Sciences, Khon Kaen University, Khon Kaen 40002, Thailand; 6 Integrative Pharmaceuticals and Innovation of Pharmaceutical Technology Research Unit, Faculty of Pharmacy, Mahasarakham University, Maha Sarakham 44150, Thailand

**Keywords:** germination, illumination, melatonin, polyphenols, tryptophan, sprouts

## Abstract

Mung bean (*Vigna radiata* L.) sprouts are increasingly consumed and have become part of a healthy diet. The sprouts are composed of proteins, carbohydrates, and biochemical compounds. During germination, the phytochemical compounds are significantly elevated, especially under stress conditions such as salinity, drought, extreme temperature, and illumination. The present study examined the effects of light and germination time on the bioactive compounds in mung bean sprout extracts. Mung bean seeds were sprouted under different light exposure conditions, and the phytochemical composition and antioxidant activity of sprout extracts were determined compared to seeds. The results show that tryptophan sharply decreased during germination. On the contrary, melatonin, polyphenols, and total phenolic content (TPC) were elevated with increased germination time, correlated with increased antioxidant activity. Sprouts germinated in the dark presented higher levels of melatonin and TPC compared with those germinated under 12 h light exposure (3.6- and 1.5-fold, respectively). In conclusion, germination can enhance valuable phytochemicals and antioxidant activity of mung bean sprouts. Mung bean sprouts may be a good alternative functional food for promoting human health.

## 1. Introduction

The consumption of sprouts has increased in recent years, due to their high nutritional value [1]. Sprouts are young seedlings obtained via seed germination with a short growth period. Germination is a process used to overcome the antinutritional or indigestible factors that limit the use of legumes for food [2,3]. During this process, these factors are decreased, while the nutritional components (e.g., glucose, amino acids, and fatty acids) and polyphenols are increased [1,2]. Moreover, seed germination under abiotic stress conditions, including salinity, drought, low temperature, and illumination, has been reported to significantly increase the phytochemical content, including phenolics, flavonoids, and melatonin [4,5,6,7]. Light exposure also influences the production of bioactive compounds. Numerous phytochemical compounds are increased when seeds are germinated in the dark. The total phenolic content (TPC) of mung bean sprouts and the increased melatonin (indolamine compound) content in kidney bean and lentil sprouts are related to their germination periods [8,9,10]. Melatonin (*N*-acetyl-5-methoxyryptamine) is a crucial hormone mainly produced in the human pineal gland that plays an important role in circadian rhythm. It is also associated with growth hormone secretion in children [11]. Additionally, melatonin regulates energy metabolism and the immune system [12] and possesses various biological activities, such as anti-inflammatory [13], antioxidant [14], and neuroprotective [15] activity. The nocturnal plasma level of melatonin increases to reach a maximum at the age of 1–3 years. Thereafter, the concentration gradually declines with increasing age [16]. Supplementation with melatonin not only delays age-related impairments but also improves physical, physiological, and psychological capabilities [17]. The biosynthesis pathway of melatonin initiates from the biosynthesis of tryptophan, an amino acid, and is stimulated by darkness and inhibited by light. Besides humans, melatonin is also found in other vertebrates, as well as bacteria, algae, and plants [18]. As a plant hormone, it affects growth promotion, such as rooting induction and germination, as well as photosynthesis [19]. The scientific evidence shows that the consumption of plants enriched with melatonin or tryptophan can increase serum melatonin levels [6,20,21,22]. Since melatonin has been reported to play an important physiological role in humans, the potential effect derived from exogenous melatonin in plant-based foods or dietary supplements for human use should be investigated further.

In addition, the increase in phytochemicals also improves the antioxidant capacity [6]. The germination period and abiotic conditions stimulate the phytochemical content of plant seeds; therefore, a suitable germination period and conditions will increase some nutritional and antioxidant phytochemicals to promote mung bean sprouts as a good source of enriched antioxidant agents. However, the polyphenol and endonitrogenous contents of mung bean seeds have not been quantified with regard to germination period and illumination. The aim of this study was, therefore, to investigate the effects of the germination process and illumination on the variety and content of phytochemical compounds with antioxidant activity (melatonin, tryptophan, phenolics, and flavonoids) as an alternative way to increase the essential nutrients of plant food as well as improve consumer health.

## 2. Results and Discussion

### 2.1. Effect of Germination and Illumination on Phytochemical Components of Mung Bean Sprouts

#### 2.1.1. Determination of Melatonin and Tryptophan Content

**Figure 1:**
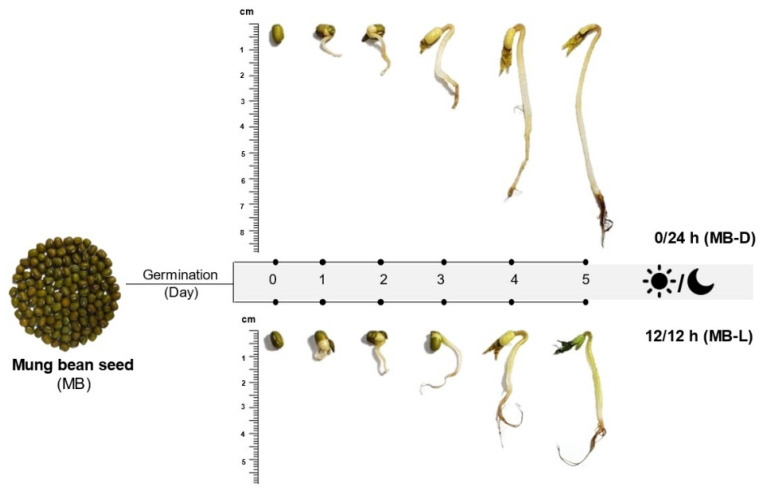
Appearance of mung bean (MB) seeds and sprouts in the dark (0/24 h; MB-D) and illumination (12 h/12 h; MB-L) on day 0 to day 5.

Different germination conditions resulted in different appearances of mung bean sprouts, as shown in Figure 1. Briefly, the germination could be divided into two stages. The first stage, water imbibition, caused the seed hull to split, and the radicles emerged after 2 days of germination. During the second stage, the sprout was growing and resulted in the emergence of hypocotyl, foliage leaves, and secondary roots. While growing, the sprouts that were exposed to the light developed green foliage leaves, while the dark germination sprouts had yellow leaves. For sprouts in complete darkness (0/24 h), the saplings were more elongated compared to those germinated in 12 h/12 h illumination. The elongation of sprouts germinated in the dark may be due to changes in the biosynthesis of secondary metabolites, such as melatonin, which has been reported to be a plant growth promoter [23,24]. The chemical structure of melatonin is similar to that of the phytohormone auxin, namely indole-3-acetic acid (IAA), which promotes the elongation of plants [19].

**Figure 2:**
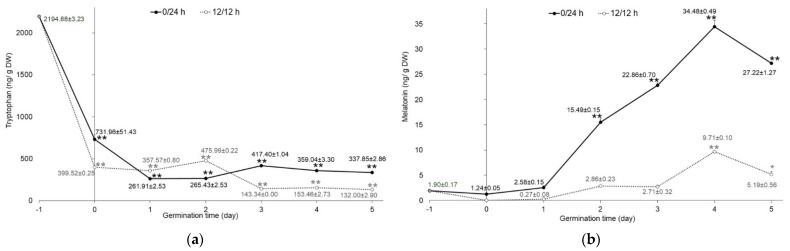
Content of indole compounds in germinated mung beans: (**a**) tryptophan and (**b**) melatonin. Mung beans were germinated in complete darkness (0/24 h; black line) and under illumination (12 h/12 h; dotted line) from day 0 to 5. Data of day−1 refer to the raw material of mung seeds, as mentioned above (*n* = 3). Error bar represents SD; * *p* < 0.05, ** *p* < 0.001.

Tryptophan, serotonin, and melatonin content was determined in mung bean sprouts germinated in the dark (0/24 h) and under illumination (12 h/12 h). Quantitative analysis by HPLC-FD showed that only tryptophan and melatonin were found in the extracts. Serotonin, which is an intermediate in the biosynthetic pathway of melatonin in plants, was not detected in this study. The tryptophan and melatonin content of mung bean sprouts germinated in the dark was significantly higher (*p <* 0.001) compared to sprouts germinated with 12 h/12 h illumination. Nevertheless, the altered concentration of tryptophan in both germination conditions presented the same trend. Raw mung bean seeds had the highest amounts of tryptophan (2194.88 ± 3.23 ng/g DW). The tryptophan levels in soaking samples were significantly decreased (*p <* 0.001), by about three- to five-fold, compared with the raw seeds, as shown in Figure 2a. As the germination time progressed, tryptophan levels exhibited a slight decrease. This may be attributed to the biosynthesis of melatonin during germination. Tryptophan is an essential amino acid in plants and serves as a precursor for the biosynthesis of melatonin [19]. On the other hand, the results from Van Hung et al. [9] showed that tryptophan was not found in mung bean seeds and sprouts germinated in both darkness and light conditions for 4 days.

By contrast, melatonin in sprouts significantly increased (*p* < 0.001) from the first to the fifth day of germination compared with raw seeds (1.90 ± 0.17 ng/g DW), as shown in Figure 2b. The concentration of melatonin reached a peak of 34.48 ± 0.49 and 9.71 ± 0.10 ng/g DW on the fourth day of germination in darkness (0/24 h) and illumination (12 h/12 h), respectively. Sprouts germinated in the dark had higher melatonin levels (one- to nine-fold) than those germinated under illumination. These results correspond to the increased melatonin levels in lentil sprouts with more germination time [10,25]. In the biosynthesis of melatonin, starting with the essential precursor, tryptophan, it undergoes many conversions by the enzymes involved and, finally, obtains melatonin. The final two enzymes in melatonin’s biosynthetic pathway, serotonin *N*-acetyl transferase (SNAT) and hydro-xyindole-O-methyltransferase (HIOMT), which catalyze *N*-acetylation of serotonin and O-methylation of *N*-acetyl serotonin to generate melatonin, were influenced by the exposure of the light. Melatonin levels of rice seedlings were higher in the dark conditions [26].

#### 2.1.2. Changes in Total Phenolic and Flavonoid Contents

**Figure 3:**
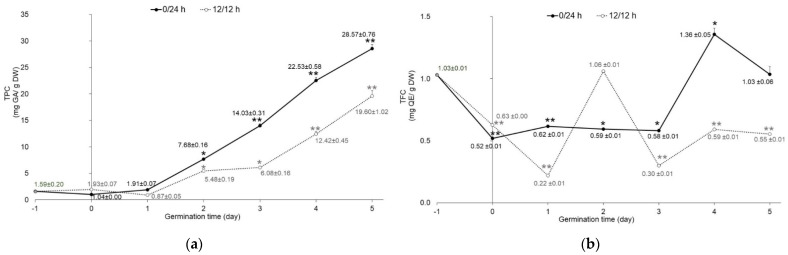
Content of (**a**) Total phenolic content (TPC) and (**b**) (TFC) total flavonoid content (TFC) of mung beans germinated in complete darkness (0/24 h; black line) and illumination (12 h/12 h; dotted line) from day 0 to day 5. Data of day−1 represent raw material of mung seeds (*n* = 3). Error bar represents SD; * *p* < 0.05, ** *p* < 0.001.

The total phenolic content of mung bean seeds was 1.59 ± 0.20 mg gallic acid equivalent (GA)/g dry weight (DW). The germination process and illumination conditions (12 h/12 h and 0/24 h light/dark periods) affected the total phenolic content (TPC), as shown in Figure 3a. Compared with raw seeds, the TPC of sprouts significantly increased in a time-dependent manner in both conditions and, on day 5, reached a peak of 28.57 ± 0.76 and 19.60 ± 1.02 mg GA/g DW in darkness and illumination, respectively. The trend of TPC change was in agreement with previous studies on mung bean seeds. Germinated mung bean seeds had higher TPC than raw seeds [5]. The previous studies reported that the important defensive response of sprout survival during germination is phenolic biosynthesis. Phenolic compounds were found as free and bound forms in the cell walls of the plants. The germination process would release the bound phenolics by enzyme hydrolyzation and made them easily solubilized, resulting in a change to the ratio of free and bound phenolics [8,9,27]. Regarding the effect of light exposure on TPC, sprouts germinated in the dark presented higher TPC than those exposed to light (Figure 3a). A similar trend was observed in other seed sprouts, such as kidney bean [4], alfalfa seed, chicory seed, rapeseed, red kale seed, and sunflower seed [28]. Many researchers have reported that germination under dark conditions resulted in improved secondary metabolites of plant seeds [4,9,10]. Our results agree with these reports, as the mung bean sprouts had an improved content of total phenolic compounds.

In terms of TFC, soaked mung bean seeds at day 0 had dramatically reduced (*p* < 0.001) TFC compared to raw seeds in both illumination conditions. The 12 h/12 h light/dark germination resulted in significantly decreased (*p* < 0.001) TFC, reaching the lowest level at day 1 of germination. Then, it increased to the peak level at day 2. After that, TFC fluctuated from day 3 to day 5 (Figure 3b). Sprouts germinated in the dark exhibited the same pattern as sprouts germinated under 12 h/12 h light/dark. TFC reached a peak at day 4 and was at a higher value than in raw mung bean seeds. Our results show that sprouts germinated in the dark had higher TFC than those germinated under 12 h/12 h light/dark. Previous studies reported that the TFC of mung beans increased during germination under white LED and UV lights [9,29]. Flavonoids were evaluated as sunscreen for plants to protect them from UV exposure by increased biosynthesis via increased phenylalanine ammonia-lyase (PAL) and flavonoid biosynthesis genes [30]. UV-A has been reported to induce specific organ flavonoids in the roots of soybean sprouts [31]. It is well known that several flavonoids such as anthocyanin and catechins can dissolve in water. This contributes towards the loss of flavonoids due to water absorption during germination. Therefore, the decreased TFC value in this study can be attributed to water absorption, light, and oxygen, which occur during the germination process. Moreover, differences in plant varieties and extraction methods also affect TFC value [32]. In this study, soaking did not have a positive effect on mung bean seeds under both illumination conditions, which may be due to the decrease in anti-nutrients (carbohydrates, proteins, etc.), thus losing the soluble or bound phenolics, which are mainly polyphenols [3,33]. It is hypothesized that at the initial stage of germination, carbohydrates and proteins are degraded, accompanied with the increase in the bound phenolics conjugated with the cell wall components. With the germination time increase, the new bound phenolics were synthesized and secreted to the cell wall. Therefore, germinated sprouts can be an alternative natural source for providing polyphenol compounds.

#### 2.1.3. Identification and Quantification of Phenolic and Flavonoid Compounds in Mung Bean Seeds and Sprouts

Phenolic and flavonoid compounds were identified and quantified by HPLC-UV-DAD via the standard compounds of the phenolic acid (group of hydroxybenzoic and hydroxycinnamic acids) and flavonoids. Six phenolic acid compounds were identified in mung bean seeds and sprouts: four hydroxybenzoic acid compounds, gallic acid (GA), *p*-hydroxybenzoic acid (*p*-HO), protocatechuic acid (PCCA), and vanillic acid (VA); two hydroxycinnamic acid compounds, *p*-coumaric acid (*p*-CA) and ferulic acid (FA); and two flavonoid compounds, rutin (RN) and quercetin (QE) (Table 1).

The previous study was the first investigation of the effect of illumination on the changes in phenolic profiles of mung bean sprouts during germination. The soaking process did not affect the phenolic compounds, which were similar to TPC and TFC. Only four compounds were identified in mung bean seeds, in smaller amounts: GA, *p*-HO, RN, and QE, the major polyphenols found in mung bean seeds [34]. On the other hand, the germination process affected the qualification and quantification of phenolic and flavonoid compounds in mung beans according to germination time. During the germination process under dark and light conditions, along with the four compounds found in raw seeds, PCCA, VA, *p*-CA, and FA were also found. Mung bean sprouts germinated in complete darkness (0/24 h) had the highest amounts of GA (12.85 ± 0.13 µg/g DW), PCCA (8.57 ± 0.14 µg/g DW), and FA (3.36 ± 0.13 µg/g DW), but sprouts germinated under 12 h/12 h light/dark presented the highest amounts of *p*-HO (10.80 ± 1.55 µg/g DW), VA (7.37 ± 2.09 µg/g DW), and *p*-CA (11.36 ± 2.48 µg/g DW). The amounts of phenolic compounds were significantly higher in the last germination period, except FA and RN, which were higher in the initial period (Table 1). Moreover, the increased phenolic compounds correlated with TPC in both conditions, thus, the germination process affects the increasing trend of polyphenol compounds in sprouts over time [35,36]. Polyphenols in plants are normally synthesized in response to harsh environments, such as soil salinity, drought, and extreme heat and light conditions [37]

The determination of flavonoids (RN and QE) in this study is accordance with a previous study reporting quantification of RN and QE in mung bean sprouts grown for 3 days [38]. Meanwhile, in this study, the amounts of RN and QE in mung bean sprouts fluctuated under both conditions, similar to TFC, and the amount of RN in mung bean sprouts did not depend on light or dark conditions. The biosynthesis of flavonoids’ compounds depends on light, and the highest amounts are mostly found in the leaves and external parts of plants, which are the parts illuminated by light [39].

### 2.2. Effect of Germination and Illumination on Antioxidant Capacity of Mung Bean Seeds and Sprouts

The results show that the germination process had a positive effect on the antioxidant activity of mung bean sprouts by increasing over time, according to ABTS, DPPH, and FRAP assays (Table 2).

Soaked mung bean seeds both with and without illumination did not show a significant increase in antioxidant levels. A possible reason for the reduction in antioxidant activity may be related to the reduction in the amount of polyphenol compounds, as the soaking process is the state of nutritional preparation for plant growth [33]. In the germination process, antioxidant activity in terms of ABTS^•+^ and DPPH^•^ radical scavenging showed a significant increase (*p <* 0.05) from the first day of the germination period under controlled illumination conditions, both with and without light. During the initial germination phase, the antioxidant levels did not show a consistent trend, then significantly increased from day 3 by 2- to 3-fold for ABTS^•+^ radical-scavenging and 1.46- to 1.82-fold for DPPH^•^ radical scavenging. FRAP assay displayed increasing Fe^2+^ ion chelation during the germination process, which was not statistically significant (*p <* 0.05). Our results agree with those of a study by Van Hung et al. [9], which showed increasing antioxidant activity in mung beans during germination over time. Reactive oxygen species (ROS) accumulation may also be beneficial for seed germination and seedling growth [40,41]. The trend of increasing antioxidant activity correlates with that of antioxidant phenolic compounds. Phenolic compounds also provide essential functions in plant growth. Bound phenolics can be synthesized and secreted from the cell wall during germination periods. Mung bean seed contained both free phenolic and bound phenolics in the ratios of 80% and 20%, respectively. The germination changed the content of free phenolic compounds to 95% and 5% of bound phenolic [8]. In summary, the germination dramatically increased total phenolic compounds in a time-dependent manner. Therefore, the germination of mung bean sprouts dramatically increased polyphenols and antioxidant activity, when compared to those of mung bean seeds.

### 2.3. Correlation of Phytochemical Compounds and Antioxidant Capacity of Mung Beans Based on the Effect of Germination under Different Illumination Conditions

**Figure 4:**
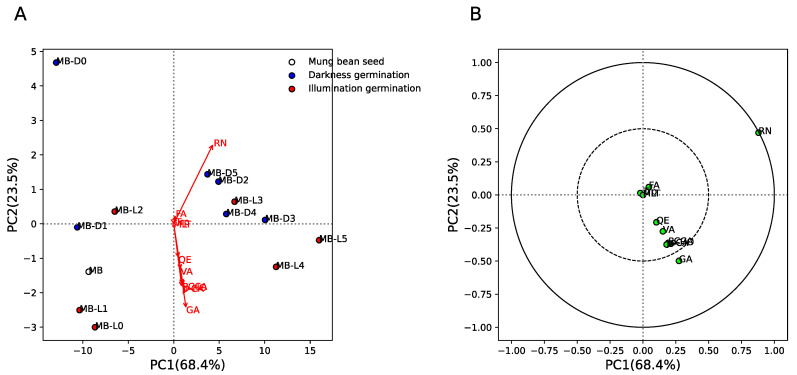
Principal componence analysis (PCA) of phytochemical components between mung bean seeds and sprouts germinated in darkness (0/24 h; MB-D0 to MB-D5) and illumination (12 h/12 h; MB-L0 to MB-L5) for 5 days. (**A**) Score plot of MB, MB-D0 to MB-D5, and MB-L0 to MB-L5. White, blue, and red colors indicate MB, MB-D, and MB-L, respectively. Red arrow indicates the phytochemical components of MB, MB-D0 to MB-D5, and MB-L0 to MB-L5. (**B**) Loading plot of phytochemical components of MB, MB-D0 to MB-D5, and MB-L0 to MB-L5. Green circles indicate the phytochemical components of MB, MB-D0 to MB-D5, and MB-L0 to MB-L5. Dashed and solid lines indicate correlation >0.5 and 1, respectively.

Figure 4 shows the principal component analysis (PCA) results of phytochemical components between mung bean seeds and sprouts in different germination conditions (darkness, MB-D0 to MB-D5, and illumination, MB-L0 to MB-L5). The results show that for mung bean seeds, early days of germination in the dark (MB-D0 and MB-D1) and under illumination (MB-L0, MB-L1, and MB-L2) were separated from late days in darkness (MB-D2 to MB-D5) and under illumination (MB-L3 to MB-L5) by PC1 (explaining 68.4% of variance) and PC2 (explaining 23.5% of variance) (Figure 4A). This indicates similar quantities of phytochemical components in the early and late days of germination. Figure 4B shows the loading plot between phytochemical components and their respective loading scores. The results show that rutin (RN) and gallic acid (GA) were strongly positively correlated with PC1 (late days of germination in darkness and illumination). This result indicates that RN and GA are increased in the late days, regardless of the darkness/illumination conditions. However, we found that MB-D2 to MB-D5 could be separated from MB-L4 and MB-L5 by the GA component, which means GA can also be used to classify darkness/illumination conditions at the late stage of the germination process.

**Figure 5:**
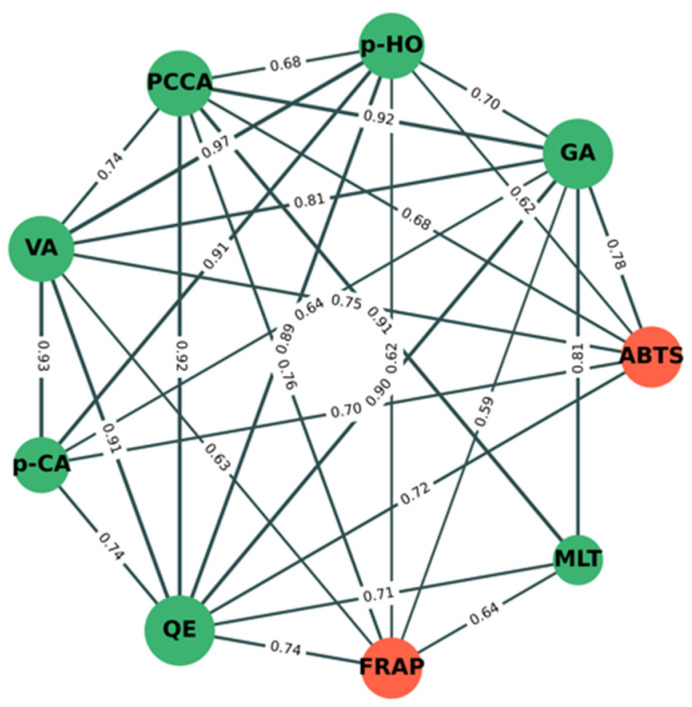
Correlation network analysis of phytochemical components and antioxidant activity between mung bean seeds and mung bean sprouts under different conditions. Green and orange nodes indicate phytochemical components and antioxidant activity, respectively. Correlation coefficient values (*r*) between nodes are shown on green lines. Edge (line) thickness indicates strength of correlation between node pairs.

Figure 5 shows the significant correlation (*p <* 0.05) network analysis of phytochemical components (green nodes) and antioxidant activity (orange nodes) between mung bean seeds and mung bean sprouts under different conditions. The results show that percentage inhibition by ABTS was strongly positively correlated with the amounts of GA (*r* = +0.78), VA (*r* = +0.75), QE (*r* = +0.72), *p*-CA (*r* = +0.70), PCCA (*r* = +0.68), and *p*-HO (*r* = +0.62). This indicates that increasing amounts of GA, VA, QE, *p*-CA, PCCA, and *p*-HO may directly correlate with inhibitory activity by ABTS. The FRAP value was also strongly positively correlated with the amounts of PCCA (r = +0.76), QE (*r* = +0.74), MLT (*r* = +0.64), VA (*r* = +0.63), *p*-HO (*r* = +0.62), and GA (*r* = +0.59). This indicates that increasing amounts of PCCA, QE, MLT, VA, *p*-HO, and GA may directly correlate with inhibitory activity by FRAP. PCCA, QE, VA, *p*-HO, and GA have a direct correlation with both antioxidant assays, whereas *p*-CA and MLT have a direct correlation with ABTS and FRAP, respectively. Indirectly, *p*-CA and MLT were strongly positively correlated with PCCA, QE, VA, *p*-HO, and GA, confirming that both compounds were also correlated with both antioxidant assays.

Interestingly, we found that the inhibitory activity of mung bean seeds and sprouts by DPPH was not significant correlated with the nine phytochemical components evaluated in this study. Taking these results together, it suggests that only gallic acid strongly contributes to the separation between early and late stages of germination, the separation between the late stage of germination in darkness and illumination (Figure 4), and the antioxidant activity of mung bean seeds and sprouts (Figure 5).

## 3. Materials and Methods

### 3.1. Chemicals and Reagents

All chemical reagents used in this study for extraction and analysis were of analytical grade. For antioxidant assays, 2,2′-azinobis (3-ethylbenzothiazoline-6-sulfonic acid) disodium salt (ABTS), 2,2-diphenyl-1 picrylhydrazyl (DPPH) radical, potassium persulfate, 2,4,6-tris(2-pyridyl)-1,3,5-triazine (TPTZ), 2,2′-azobis(2-methylpropionamidine) dihydrochloride, and aluminum chloride hexahydrate were purchased from Sigma Aldrich (St. Louis, MO, USA). Iron (III) chloride was purchased from Ajax Finechem (New South Wales, Australia), sodium carbonate from Kemaus (New South Wales, Australia), and Folin–Ciocalteu reagent from LOBA Chemis (Mumbai, India). HPLC standards for tryptophan and serotonin, gallic acid (GA), *p*-hydroxybenzoic acid (*p*-HO), protocatechuic acid (PCCA), vanillic acid (VA), *p*-coumaric acid (*p*-CA), ferulic acid (FA), rutin (RN), and quercetin (QE) were purchased from Sigma Aldrich (St. Louis, MO, USA). HPLC standard for melatonin was purchased from Shanghai Chemical (Shanghai, China). All organic solvents, including AR grade and HPLC grade, were purchased from ACI Labscan (Bangkok, Thailand).

### 3.2. Preparation of Mung Bean Germination

The mung beans (*Vigna radiata* L. Wilczek) used in this study were purchased from the Khon Kaen local market in Thailand. Three batches of mung bean seeds (20 g/batch) were soaked in warm (60 °C) water and kept at room temperature for 8 h. This was designated as day 0. After removing the water, the soaked seeds were germinated at room temperature for 5 days under the following dark–light conditions: 12 h/12 h (light/dark) and 0/24 h (complete darkness). Seeds germinating in the dark (0/24 h) were covered with a black plastic bag from day 1 to day 5. An LED source was used for the 12 h/12 h illumination condition. Samples were taken from the container each day, dried, and stored at −20 °C for further study. The appearance of the germinated mung bean seeds is shown in Figure 1.

### 3.3. Mung Bean Sprout Extraction

For the extraction process, 20 g of mung bean seeds with sprouts was pulverized and suspended in 200 mL methanol (1:10 *w*/*v*) and ultrasonicated for 30 min at room temperature [40], which was controlled at ultrasonic frequency of 28 KHz and 220 Volts by Ultrasonic cleaners. The extraction was repeated three times. Organic layers were combined and dried over a rotary evaporator to yield methanol extract and kept at −20 °C until further experimental use.

### 3.4. Determination of Tryptophan, Serotonin, and Melatonin by HPLC-FD

HPLC-FD analysis of tryptophan, serotonin, and melatonin was adapted from Chen et al. [42]. Samples were reconstituted for solid-phase extraction (SPE; VertiPack C18 Tubes 500 mg/3 mL). First, SPE was activated with 10 mL of methanol and 10 mL of distilled water, respectively. After that, 2 mL of sample solution (50 mg of the sample with 5% methanol) was eluted with 10 mL of 5% methanol. Finally, 5 mL of 80% methanol was added, and the supernatants were kept dry under N_2_. The residues were dissolved in a mixture of MeOH and 50 mM phosphate buffer at pH 4.5 (1:1; *v*/*v*). Then, 20 μL of each sample concentration was injected. This assay was performed using automated injection on an Agilent 1260 system (Agilent, California, USA) consisting of quaternary pumps with Unisol C18 5 µm 4.6 × 250 columns, with a flow rate of 1 mL/min and excitation and emission wavelengths of 286 and 346 nm. Mixtures of methanol (solvent A) and 50 mM phosphate buffer at pH 4.5 (solvent B) as the mobile phase were pumped into the column with the following gradients: 25% solvent A (0–15 min), 40% solvent A (15–45 min), and 25% solvent A (45–60 min). Quantification was performed using the pure standards of tryptophan, serotonin, and melatonin. Samples and standard solutions were analyzed in triplicate. The concentrations of tryptophan, serotonin, and melatonin are shown as ng/g dry weight (DW). The analytical method was validated according to the international council for harmonization of technical requirements for pharmaceuticals for human use (ICH) guideline. The values of the limit of detection (LOD) and limit of quantification (LOQ) were 0.0025 µg/mL and 0.05 µg/mL, respectively (Table S1 and Figure S1).

### 3.5. Antioxidant Capacity

The 2,2-Diphenyl-1-picrylhydrazyl (DPPH) radical scavenging capacity assay:

To determine radical scavenging capacity, we used a DPPH assay method adapted from Arabshahi-Delouee and Urooj [43]. The reaction used a mixture of 100 µL of 200 µM DPPH and 100 µL solution of sample extract or Trolox standard (final concentration 50 µg/mL), which was incubated in the dark for 30 min. Methanol was used as a control. The absorbance of the mixture was measured at 517 nm. The percentage inhibition of DPPH of samples and a known solution of Trolox was calculated by Equation (1):

%inhibition = ((A_contol_ − A_sample_)/A_control_) × 100 (1)

where A_control_ is the absorbance of the control (517 nm), and A_sample_ is the final absorbance of the sample extract (517 nm). Results are indicated as the percentage of inhibition.

For the 2,2′-azinobis (3-ethylbenzothiazoline-6-sulfonic acid) disodium salt (ABTS) assay, the method was adapted from Brand-Williams et al. [44]. The stock solution of ABTS^·+^ oxidant agent was prepared via reaction of 7 mM ABTS^·^ with 2.45 mM potassium persulfate (K_2_S_2_O_8_) (1:1: *v*/*v*), followed by incubation in the dark for 8–12 h. After that, an aliquot (1 mL) of the stock solution of the ABTS^·+^ oxidant agent was added to 15 mL of distilled water as the ABTS^·+^ reagent, which was prepared daily.

To determine antioxidant capacity, 100 µL of ABTS^+^ reagent and 100 µL of solution of sample extract or Trolox standard (final concentration 50 µg/mL) were reacted, followed by incubation in the dark for 30 min. The absorbance of the mixture was measured at 415 nm. Methanol was used as a control. The percentage inhibition of ABTS of samples and a known solution of Trolox was calculated by Equation (1), with A_control_ = 415 nm and A_sample_ = 415 nm. The results are shown as the percentage of inhibition.

For the ferric reducing antioxidant power (FRAP) assay, the method followed Iqbal et al. [45]. The FRAP reagent was prepared with 3 mixing solutions: 300 mM acetate buffer (pH 3.6), 20 mM ferric chloride solution, and 10 mM 2,4,6-tris (2-pyridyl)-1,3,5-triazine (TPTZ) solution (10:1:1; *v*/*v*/*v*).

For antioxidant capacity determination, the FRAP reagent and sample extract solution (80:20; *v*/*v*) were reacted, then incubated in the dark for 5 min, and absorbance was read at 595 nm. Methanol was used as a blank. Quantification was performed using the known solution, ferrous sulfate (FeSO_4_·7H_2_O). The antioxidant capacity results are expressed as Fe^2+^ mmole/100 g DW.

### 3.6. Total Phenolic Content

The method to determine total phenolic content (TPC) was modified from the method of Um et al. [46]. A reaction mixture containing 20 µL of sample extract solution, 80 µL of 7% sodium carbonate (Na_2_CO_3_), and 100 µL of 10% Folin–Ciocalteu reagent was incubated for 30 min in the dark. Absorbance was measured at 760 nm using a microplate photometer (Sunrise TM, Grödig, Austria). Quantification was analyzed from the standard curve of gallic acid standard (1–50 µg/mL). The results are expressed as mg of gallic acid equivalent per gram of dry weight (mg GA/g DW).

### 3.7. Total Flavonoid Content

The method of assaying total flavonoid content (TFC) was modified from Čopra-Janićijević et al. [47]. The assay was carried out by mixing sample extract and 2% aluminum chloride (AlCl_3_) (1:1; *v*/*v*), which were incubated for 30 min in the dark. Methanol was used as a blank, and absorbance was recorded at 415 nm. Qualification was performed by using a quercetin standard curve at concentrations of 1 to 20 µg/mL. The results are expressed as mg of quercetin equivalent per gram dry weight (mg QE/g DW) of sample.

### 3.8. Quantitative and Qualitative Determination of Phenolic Compounds by HPLC-UV-DAD

The HPLC quantitative analysis of phenolic compounds was adapted from Kaisoon et al. [48]. Determination of phenolic compounds was performed using a Shimadzu instrument (Shimadzu Co., Kyoto, Japan) with a diode array detector, and chromatographic separations were performed on a Unisol C18 column (4.6 × 250 mm, 5 µm). The mobile phase consisted of 1% acetic acid (solvent A) and 100% acetonitrile (solvent B) at a flow rate of 0.8 mL/min. The gradient elution program started with 5% solvent B for 5 min followed by 9% solvent B from 5 to 15 min, 11% solvent B from 15 to 22 min, 18% solvent B from 22 to 38 min, 23% solvent B from 38 to 43 min, 90% solvent B from 43 to 44 min, 80% solvent B from 44 to 45 min, 80% solvent B from 45 to 55 min, isocratic, 5% solvent B from 55 to 65 min, re-equilibration, and 5% solvent B from 65 to 70 min, linear gradient. Phenolics and flavonoids were identified as equivalents of the aforementioned standards. The analytical method showed the LOD and LOQ of phenolic acids as 0.001 µg/mL and 0.01 µg/mL, respectively (Table S2 and Figure S2).

### 3.9. Statistical Analysis

Values were statistically analyzed using one-way analysis of variance (ANOVA) followed by Tukey’s post hoc test (SPSS version 26; SPSS Inc., Armonk, New York, USA). Results were considered significant at a *p*-value of <0.05 (*n* = 3).

Principal component analysis (PCA) of phytochemical components between mung bean seeds and sprouts was performed by using Python software version 3.10.5 (Python Software Foundation, Fredericksburg, Virginia, USA). The phytochemical components were normalized before eigenvalues were calculated and then fit transformed into PC scores. The PCA scores and loading variables were obtained from the model. A PCA score plot was used to illustrate the phytochemical distribution between mung bean seeds and sprouts in different conditions. The separation loading variable between seeds and sprouts is shown in the correlation plot, with the correlation circle at 0.5 (moderate correlation) and 1.0 (strong correlation).

Correlation-based network analysis of phytochemical components and antioxidant activity between mung bean seeds and sprouts was performed by using Python version 3.10.5 and NetworkX software version 2.8.4. The correlation analysis was performed using Pearson’s correlation. The results were considered statistically significant at a *p*-value of <0.05. Only statistically significant correlations (*p* < 0.05) were used to construct the network. Green and orange nodes represent phytochemical components and antioxidant activity, respectively. The edge thickness indicates Pearson’s correlation coefficient values (*r*); *r* < 0.5 indicates a weak correlation, while *r* > 0.5 indicates a strong correlation. The green edge represents the positive correlation between each node pair.

## 4. Conclusions

The germination conditions, including the light exposure condition and germination time, significantly influenced the changes in phytochemical composition and functional properties of mung bean seeds. The results show that the melatonin, gallic acid, *p*-hydroxybenzoic acid, protocatechuic acid, vanillic acid, *p*-coumaric acid, and quercetin contents and antioxidant activity increased during germination. In addition, tryptophan content showed a decreasing trend during germination. Melatonin and tryptophan contents of mung bean sprouts germinated in the dark (0/24 h) were found to be higher than in sprouts germinated under illumination (12 h/12 h). Positive correlations were observed between melatonin and *p*-coumaric acid content and antioxidant activity by ABTS and FRAP assay. In conclusion, mung bean sprouts germinated with and without light have increased phytochemical compounds and antioxidant activity compared to raw seeds. Nowadays, people are encouraged to increase the consumption of plant-derived foods to improve their health. Mung bean sprouts would be considered as an excellent source of bioactive compounds with high antioxidant activity. In addition, the study of mung bean seed germination might be utilized as a model for nutraceutical improvement to other plant produces.

## Figures and Tables

**Table 1:** Phenolic profile of mung bean (MB) seeds and sprouts germinated in darkness (0/24 h) (MB-D0 to MB-D5) and illumination (12 h/12 h) (MB-L0 to MB-L5) for 5 days.

Sample	Germination Time (Day)	Phenolic Compounds (µg/g DW)							
		GA	*p*-HO	PCCA	VA	*p*-CA	FA	RN	QE
MB	-	0.26 ± 0.00 ^g^	0.75 ± 0.01 ^e^	NQ	NQ	ND	ND	32.36 ± 0.71 ^de^	1.36 ± 0.00 ^d^
MB-D	0	0.19 ± 0.01 ^g^	0.36 ± 0.01 ^e^	NQ	NQ	NQ	0.68 ± 0.10 ^de^	37.58 ± 3.59 ^bcde^	0.50 ± 0.03 ^f^
	1	0.38 ± 0.00 ^g^	0.63 ± 0.01 ^e^	0.05 ± 0.00 ^e^	NQ	NQ	1.27 ± 0.03 ^cd^	50.14 ± 1.03 ^ab^	0.76 ± 0.01 ^ef^
	2	3.57 ± 0.20 ^e^	0.84 ± 0.01 ^e^	2.47 ± 0.56 ^cd^	1.11 ± 0.05 ^cd^	NQ	3.36 ± 0.13 ^a^	42.43 ± 1.28 ^abcd^	1.53 ± 0.01 ^c^
	3	6.30 ± 0.04 ^cd^	1.49 ± 0.02 ^de^	4.84 ± 0.20 ^b^	1.47 ± 0.08 ^cd^	0.93 ± 0.06 ^cd^	3.10 ± 0.11 ^a^	46.18 ± 1.23 ^abc^	2.43 ± 0.02 ^c^
	4	12.85 ± 0.13 ^a^	3.71 ± 0.07 ^c^	8.20 ± 0.24 ^a^	3.12 ± 0.19 ^bc^	2.20 ± 0.16 ^bcd^	1.88 ± 0.17 ^bc^	47.74 ± 1.48 ^abc^	4.16 ± 0.03 ^b^
	5	6.98 ± 0.11 ^c^	7.19 ± 0.07 ^b^	8.57 ± 0.14 ^a^	3.94 ± 0.20 ^b^	2.96 ± 0.13 ^bc^	0.98 ± 0.06 ^cd^	43.87 ± 0.94 ^abcd^	4.76 ± 0.03 ^a^
MB-L	0	0.26 ± 0.01 ^g^	0.49 ± 0.01 ^e^	ND	NQ	NQ	1.47 ± 0.16 ^cd^	28.38 ± 2.23 ^e^	0.84 ± 0.01 ^ef^
	1	0.47 ± 0.02 ^g^	0.83 ± 0.02 ^e^	0.03 ± 0.02 ^e^	NQ	0.03 ± 0.02 ^d^	2.93 ± 0.17 ^a^	53.20 ± 2.76 ^a^	0.61 ± 0.01 ^ef^
	2	1.56 ± 0.01 ^f^	0.91 ± 0.01 ^de^	0.57 ± 0.01 ^e^	NQ	0.08 ± 0.01 ^d^	2.52 ± 0.02 ^ab^	38.46 ± 0.25 ^bcde^	1.45 ± 0.00 ^d^
	3	2.11 ± 0.04 ^f^	0.76 ± 0.02 ^e^	1.13 ± 0.05 ^de^	0.52 ± 0.05 ^d^	0.67 ± 0.07 ^cd^	1.25 ± 0.08 ^cd^	26.25 ± 1.05 ^e^	1.03 ± 0.01 ^de^
	4	5.75 ± 0.61 ^d^	2.50 ± 0.28 ^cd^	3.39 ± 0.68 ^bc^	2.05 ± 0.54 ^bcd^	4.51 ± 0.95 ^b^	0.69 ± 0.50 ^de^	35.06 ± 7.35 ^cde^	2.46 ± 0.12 ^c^
	5	9.37 ± 0.77 ^b^	10.80 ± 1.55 ^a^	4.78 ± 0.50 ^b^	7.37 ± 2.09 ^a^	11.36 ± 2.48 ^a^	1.85 ± 0.66 ^bc^	51.19 ± 9.84 ^ab^	4.72 ± 0.48 ^a^

GA, gallic acid; *p*-HO, *p*-hydroxybenzoic acid; PCCA, protocatechuic acid; VA, vanillic acid; *p*-CA, *p*-coumaric acid; FA, ferulic acid; RN, rutin; QE, quercetin. Superscript letters indicate differences in results between MB-D and MB-L in the same column (one-way ANOVA with Tukey’s HSD, *p* < 0.05). NQ, no quantification; ND, no detection.

**Table 2:** Antioxidant capacity of mung bean seeds (MB) and sprouts germinated in darkness (0/24 h) (MB-D0 to MB-D5) and illumination (12 h/12 h) (MB-L0 to MB-L5) for 5 days.

**Sample**	**Germination Time (Day)**	**ABTS** **(%Inhibition)**	**DPPH** **(%Inhibition)**	**FRAP Value** **(mmole Fe^2+^/100 g DW)**
MB	-	12.25 ± 0.00 ^hi^	5.20 ± 0.81 ^efg^	0.60 ± 0.01 ^b^
MB-D	0	8.03 ± 0.55 ^j^	10.83 ± 0.86 ^b^	0.48 ± 0.02 ^b^
	1	10.86 ± 0.54 ^i^	6.34 ± 0.19 ^def^	0.49 ± 0.01 ^b^
	2	26.14 ± 0.31 ^ef^	9.39 ± 0.28 ^bc^	0.63 ± 0.01 ^b^
	3	31.36 ± 0.16 ^c^	8.85 ± 0.85 ^bc^	0.58 ± 0.04 ^b^
	4	27.09 ± 0.27 ^de^	8.55 ± 0.47 ^bcd^	0.65 ± 0.01 ^b^
	5	24.92 ± 0.43 ^f^	9.46 ± 0.92 ^bc^	0.82 ± 0.06 ^b^
MB-L	0	13.12 ± 0.55 ^h^	3.67 ± 0.28 ^g^	0.60 ± 0.06 ^b^
	1	11.38 ± 0.27 ^i^	3.98 ± 0.28 ^fg^	0.45 ± 0.01 ^b^
	2	14.91 ± 0.16 ^g^	7.25 ± 0.37 ^cde^	0.39 ± 0.02 ^b^
	3	27.96 ± 0.3 ^d^	9.00 ± 0.60 ^bc^	0.58 ± 0.07 ^b^
	4	32.71 ± 0.48 ^c^	7.63 ± 1.51 ^cde^	0.58 ± 0.02 ^b^
	5	37.32 ± 0.59 ^b^	8.93 ± 0.43 ^bc^	0.65 ± 0.02 ^b^
Trolox	(+) control	89.71 ± 0.28 ^a^	95.90 ± 0.00 ^a^	4649.69 ± 61.54 ^a^

Superscript letters indicate differences in results between MB-D and MB-L in the same column (one-way ANOVA with Tukey’s HSD, *p* < 0.05).

## Data Availability

Not applicable.

## Supplementary Materials

The following supporting information can be downloaded at: https://www.mdpi.com/article/10.3390/plants11212990/s1, Figure S1. Chromatogram of standard tryptophan, serotonin, and melatonin at concentration of 4 μg/mL; Figure S2. Chromatogram of standard phenolic acids at concentration of 5 μg/mL. GA: gallic acid, PCCA: protocatechuic acid; p-HO: p-hydroxybenzoic acid; ChA: chlorogenic acid, VA: vanillic acid; SyA: syringic acid; p-CA: p-coumaric acid; RU: rutin; FA: ferulic acid; SA: sinapic acid; QU: quercetin. Table S1. Validation parameters of serotonin, tryptophan, and melatonin; Table S2. Validation parameters of standard phenolic acids.
