# Supplementary material for: Effect of Germination and Illumination on Melatonin and Its Metabolites, Phenolic Content, and Antioxidant Activity in Mung Bean Sprouts
Source: Plants (Basel). 2022 Nov 6;11(21):2990. doi: 10.3390/plants11212990 (PMC9654080; doi:10.3390/plants11212990)
Supplement: Supplementary file 1 [file plants-11-02990-s001.zip › plants-1957489-supplementary.pdf]

Supplementary Material

Effect of Germination and Illumination on Melatonin and Its Metabolites, Phenolic Content, and Antioxidant Activity in Mung Bean Sprouts

Pimolwan Siriparu [1], Panyada Panyatip [2], Thanawat Pota [3], Juthamat Ratha [3], Chawalit Yongram [4], Tarapong Srisongkram [5], Bunleu Sungthong [6] and Ploenthip Puthongking [3,5,*]

[1] Master of Sciences Program in Pharmaceutical Chemistry and Natural Products, Faculty of Pharmaceutical Sciences, Khon Kaen University, Khon Kaen, 40002, Thailand
[2] Department of Pharmacognosy, Faculty of Pharmacy, Srinakharinwirot University, Nakhon Nayok, 26120, Thailand
[3] Melatonin Research Group, Khon Kaen University, Khon Kaen, 40002, Thailand
[4] Division of Cannabis Health Science, College of Allied Health Sciences, Suansunandha Rajabhat University, Samut Songkhram, 75000, Thailand
[5] Division of Pharmaceutical Chemistry, Faculty of Pharmaceutical Sciences, Khon Kaen University, Khon Kaen, 40002, Thailand
[6] Integrative Pharmaceuticals and Innovation of Pharmaceutical Technology Research Unit, Faculty of Pharmacy, Mahasarakham University, Maha Sarakham, 44150, Thailand
* Correspondence: pploenthip@kku.ac.th

1. Validation parameters of serotonin, tryptophan, and melatonin

Table S1. Validation parameters of serotonin, tryptophan, and melatonin

Parameters	Serotonin	Tryptophan	Melatonin
Linear equation	y = 5.5665x + 0.0313	y = 0.9564x + 0.0138	y = 3.5414x - 0.0406
Range (µg/mL)	0.05 - 4.00	0.05 - 4.00	0.05 - 4.00
Coefficient of correlation	0.9997	0.9995	0.9998
LOD (µg/mL)	0.0025	0.01	0.0025
LOQ (µg/mL)	0.05	0.05	0.05

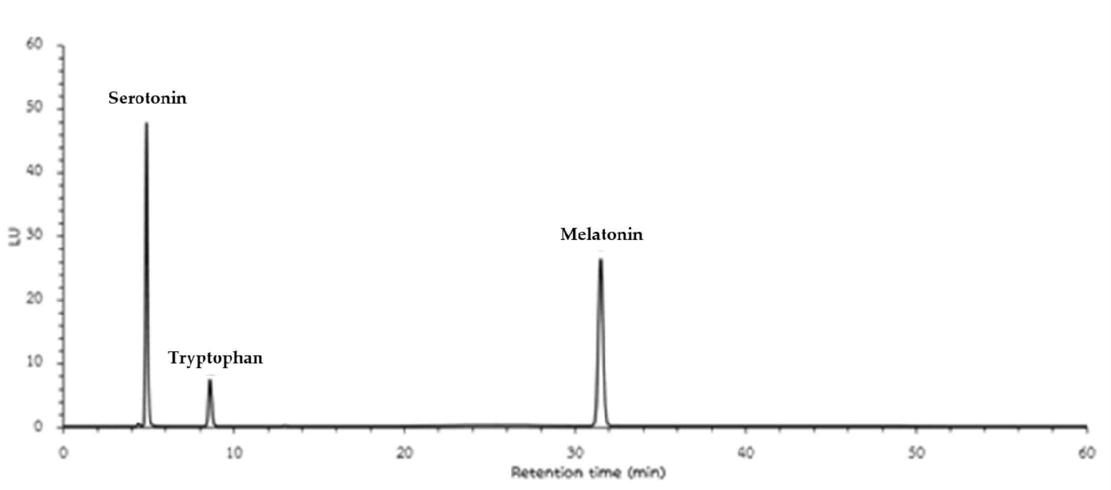

Figure S1. Chromatogram of standard tryptophan, serotonin, and melatonin at concentration of 4 µg/mL.

2. Validation parameters of phenolic acids

Table S2. Validation parameters of standard phenolic acids

Parameters	GA	PCCA	*p*-HO	ChA	VA	SyA	*p*-CA	RU	FA	SA	QU
Linear equation	y = 3647.4x + 1196.3	y = 2398.4x + 1001.9	y = 1657.9x + 775.62	y = 3027.2x + 285.11	y = 2837.3x + 1239.6	y = 5111.5x + 2274.9	y = 11200x + 3400.3	y = 2886x + 1497.7	y = 8965.9x + 2280.2	y = 8027.5x + 1552.3	y = 29567x - 28517
Range (μg/mL)	0.25 – 5.00	0.25 – 5.00	0.25 – 5.00	0.25 – 5.00	0.25 – 5.00	0.25 – 5.00	0.25 – 5.00	0.25 – 5.00	0.25 – 5.00	0.25 – 5.00	0.25 – 5.00
Coefficient of correlation	0.9998	0.9997	0.9996	1.000	0.9997	0.9997	0.9998	0.9995	0.9998	0.9999	0.9982
LOD (μg/mL)	0.005	0.025	0.025	0.025	0.010	0.010	0.005	0.025	0.010	0.005	0.001
LOQ (μg/mL)	0.025	0.100	0.100	0.100	0.100	0.025	0.010	0.100	0.025	0.025	0.025

GA: gallic acid, PCCA: protocatechuic acid; *p*-HO: *p*-hydroxybenzoic acid; ChA: chlorogenic acid, VA: vanillic acid; SyA: syringic acid; *p*-CA: *p*-coumaric acid; RU: rutin; FA: ferulic acid; SA: sinapic acid; QU: quercetin.

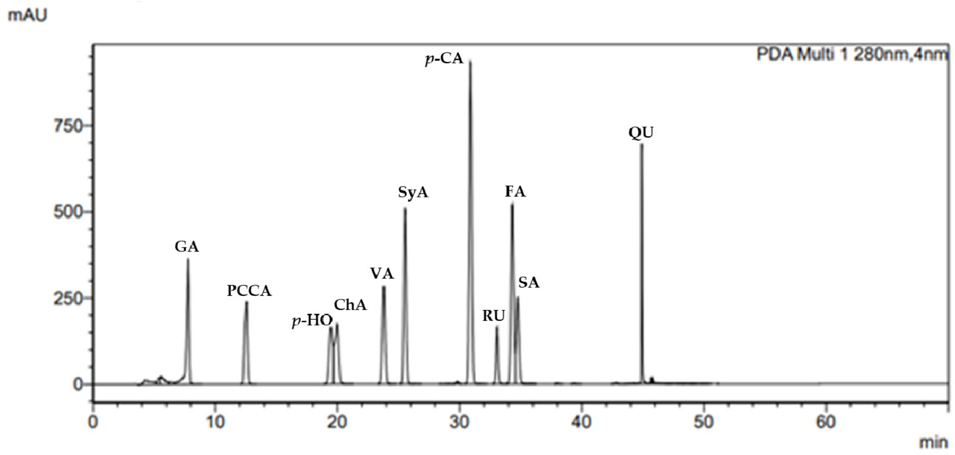

Figure S2. Chromatogram of standard phenolic acids at concentration of 5 μg/mL. GA: gallic acid, PCCA: protocatechuic acid; *p*-HO: *p*-hydroxybenzoic acid; ChA: chlorogenic acid, VA: vanillic acid; SyA: syringic acid; *p*-CA: *p*-coumaric acid; RU: rutin; FA: ferulic acid; SA: sinapic acid; QU: quercetin.
